# Long-Term Survival and Recurrence After Liver Transplantation Versus Resection in Cirrhotic Hepatocellular Carcinoma: A Systematic Review

**DOI:** 10.7759/cureus.83418

**Published:** 2025-05-03

**Authors:** Mahrukh Rehman, Maria Shah, Mehak Gul, Vanesa Shamoon, Muhammad M Tariq, Khansa Bibi, Ahmed O Ishola, Mmahaletchumy Manoharan, Nabila N Anika, Ali Bilal

**Affiliations:** 1 Surgery, S.Tentishev Asian Medical Institute, Kant, KGZ; 2 Surgery, Liaquat University of Medical and Health Sciences, Hyderabad, PAK; 3 Geriatrics, Montefiore Medical Center Wakefield Campus, New York, USA; 4 Internal Medicine, Interfaith Medical Center, New York, USA; 5 Internal Medicine, Liaquat College of Medicine and Dentistry, Karachi, PAK; 6 Internal Medicine, Foundation University Medical College, Islamabad, PAK; 7 Family Medicine, General Hospital Odan Lagos, Lagos, NGA; 8 Surgery, Universitas Sumatera Utara, Medan, IDN; 9 Surgery, Baylor College of Medicine, Houston, USA; 10 Medicine and Surgery, Holy Family Red Crescent Medical College Hospital, Dhaka, BGD; 11 Surgery, Nishtar Medical University, Multan, PAK

**Keywords:** cirrhosis, disease-free survival, hepatocellular carcinoma, liver resection, liver transplantation, long-term survival, meta-analysis, milan criteria, recurrence, systematic review

## Abstract

This systematic review evaluates and compares the long-term survival and recurrence outcomes of liver transplantation (LT) versus liver resection (LR) in patients with hepatocellular carcinoma (HCC) and underlying cirrhosis. A comprehensive search was conducted in accordance with PRISMA guidelines across PubMed, Embase, and the Cochrane Library, yielding 415 articles, of which four high-quality meta-analyses were included based on predefined eligibility criteria. The included studies encompassed tens of thousands of patients and consistently demonstrated that LT offers superior overall survival (OS) and disease-free survival (DFS), along with significantly lower recurrence rates, when compared to LR. Subgroup analyses revealed that the survival advantage of LT is more pronounced in patients within the Milan criteria or those with multifocal disease, while outcomes of LR improved over time, particularly in cases with solitary tumors and structured postoperative surveillance. The review also highlights the influence of geographic disparities, institutional practices, and healthcare resource availability on treatment selection and outcomes. Quality assessment using the AMSTAR 2 tool confirmed that three studies were of high quality with low risk of bias, while one was of moderate quality. These findings support the use of LT as the preferred treatment for eligible patients, while acknowledging LR as a valuable alternative in specific clinical scenarios, particularly where organ availability is limited.

## Introduction and background

Hepatocellular carcinoma (HCC) is the most common primary liver malignancy and ranks among the leading causes of cancer-related mortality worldwide [1]. A significant proportion of HCC cases arise in the background of chronic liver disease, particularly cirrhosis, which complicates both the management and prognosis of the disease [2]. Curative treatment options for HCC primarily include liver transplantation (LT) and liver resection (LR). While LR offers the benefit of removing the tumor without the need for organ donation, LT not only eradicates the tumor but also addresses the underlying cirrhotic liver, potentially reducing the risk of recurrence [3]. However, transplantation is limited by donor availability, perioperative risks, immunosuppressive complications, and healthcare costs.

The comparative effectiveness of LT and LR remains a matter of ongoing debate, especially in patients who meet the Milan criteria for transplant eligibility [4]: those with early-stage tumors and relatively preserved liver function. Although several studies have demonstrated superior long-term survival and lower recurrence rates with LT compared to LR, others suggest that resection remains a viable and sometimes preferable alternative in selected patients [5], particularly in regions with limited access to transplant facilities or in patients with favorable tumor biology. Furthermore, evolving surgical techniques, enhanced postoperative surveillance, and advances in LR outcomes over the past two decades warrant an updated evaluation of their comparative benefits.

Despite numerous individual studies and meta-analyses addressing this topic, variability in patient populations, study designs, surveillance protocols, and healthcare infrastructure contribute to heterogeneity in reported outcomes. This systematic review aims to synthesize the most recent and high-quality meta-analyses comparing LT and LR in cirrhotic patients with HCC, specifically focusing on long-term survival and recurrence outcomes. By doing so, it seeks to guide clinical decision-making, particularly in regions where both options are available but must be carefully matched to patient profiles.

To guide this review, a PICO framework [6] was utilized to structure the research question and ensure a focused synthesis of relevant evidence. The Population (P) of interest includes adult patients diagnosed with HCC in the setting of underlying cirrhosis, most often those who meet the Milan criteria, which define a subset of patients with early-stage tumors and preserved liver function suitable for curative interventions. The Intervention (I) examined is LT, a treatment that addresses both the malignant tumor and the diseased liver, potentially offering better long-term outcomes. The Comparison (C) involves LR, a commonly employed surgical strategy that removes the tumor but leaves the native cirrhotic liver intact, which may influence recurrence risk. The Outcomes (O) assessed in this review are long-term overall survival (OS), disease-free survival (DFS), and tumor recurrence - key indicators of treatment success in oncologic and hepatologic terms. This review aims to compare LT and LR across these critical clinical endpoints and evaluate the evolving body of evidence to help identify patient subgroups who may derive greater benefit from one modality over the other.

## Review

Materials and methods

Search Strategy

The search strategy for this systematic review was designed in accordance with the Preferred Reporting Items for Systematic Reviews and Meta-Analyses (PRISMA) guidelines [7] to ensure comprehensive and transparent identification of relevant literature comparing LT and LR in HCC patients with underlying cirrhosis. A structured search was conducted across major biomedical databases, including PubMed and Embase, using a combination of Medical Subject Headings (MeSH) and keyword terms such as “hepatocellular carcinoma”, “liver transplantation”, “liver resection”, “hepatectomy”, “cirrhosis”, “survival”, and “recurrence”. Boolean operators (AND, OR) were employed to refine the search and capture studies reporting on long-term outcomes, specifically OS, DFS, and recurrence rates. Filters were applied to include articles published in English from 2014 to 2024, focusing on meta-analyses and systematic reviews involving human subjects. Reference lists of included studies were also screened to identify any additional relevant publications. The final selection comprised four high-quality meta-analyses, each meeting predefined eligibility criteria and providing robust comparative data for synthesis.

Eligibility Criteria

The eligibility criteria for this systematic review were carefully established to include high-quality studies that directly compared long-term outcomes of LT and LR in patients with HCC and underlying cirrhosis. Only meta-analyses or systematic reviews published in peer-reviewed journals between 2014 and 2024 were considered. Included studies were required to report on at least one of the following outcomes: OS, DFS, or tumor recurrence. Studies were eligible regardless of geographical origin, provided they involved adult patients diagnosed with HCC, with a particular focus on those meeting the Milan criteria, which define early-stage disease suitable for transplantation. Both intention-to-treat (ITT) and as-treated analyses were accepted to reflect real-world treatment conditions and outcomes.

Exclusion criteria comprised case reports, narrative reviews, commentaries, single-arm studies, and studies not reporting comparative outcomes between LT and LR. Meta-analyses that focused solely on pediatric populations, non-cirrhotic HCC, or treatments other than surgical resection or transplantation were also excluded. In addition, studies lacking sufficient statistical data, such as hazard ratios, odds ratios, or clear survival percentages, were omitted to ensure consistency and quality of extracted data. This stringent selection process aimed to minimize heterogeneity and ensure that only robust, high-level evidence was included, thereby strengthening the validity and clinical relevance of the review’s conclusions.

Data Extraction

Data extraction was performed systematically using a structured format to ensure consistency and comprehensiveness across all included studies. Key information extracted from each meta-analysis included the author name, year of publication, study design, sample size, inclusion criteria or tumor stage, type of cirrhosis (if reported), type of intervention (LT), comparator (LR), follow-up duration, and primary outcomes such as OS, DFS, and recurrence rates. In addition, relevant effect measures such as hazard ratios (HRs), odds ratios (ORs), and their corresponding confidence intervals (CIs) were recorded. Qualitative observations, such as findings related to geographical variation, surveillance practices, and salvage transplantation, were also documented to capture context-specific insights. Data were reviewed and cross-checked for accuracy and completeness to minimize bias and ensure the reliability of the synthesis.

Data Analysis and Synthesis

Data analysis and synthesis were conducted narratively due to the nature of the included studies, which were all meta-analyses or systematic reviews with overlapping datasets and heterogeneous methodologies. Rather than performing a new quantitative meta-analysis, the review focused on summarizing and comparing the reported effect sizes, such as HRs and ORs, for key outcomes including OS, DFS, and recurrence rates. A thematic approach was used to highlight patterns and consistencies across studies, as well as to explore differences related to tumor stage, geographic region, surveillance protocols, and temporal trends. The strength and direction of the findings were assessed alongside the methodological quality of each study, guided by the AMSTAR 2 tool [8]. This qualitative synthesis allowed for a comprehensive and contextual interpretation of the evidence while minimizing the risk of data duplication and misinterpretation that could arise from re-pooling overlapping results.

Results

Characteristics of the Selected Studies

**Figure 1:**
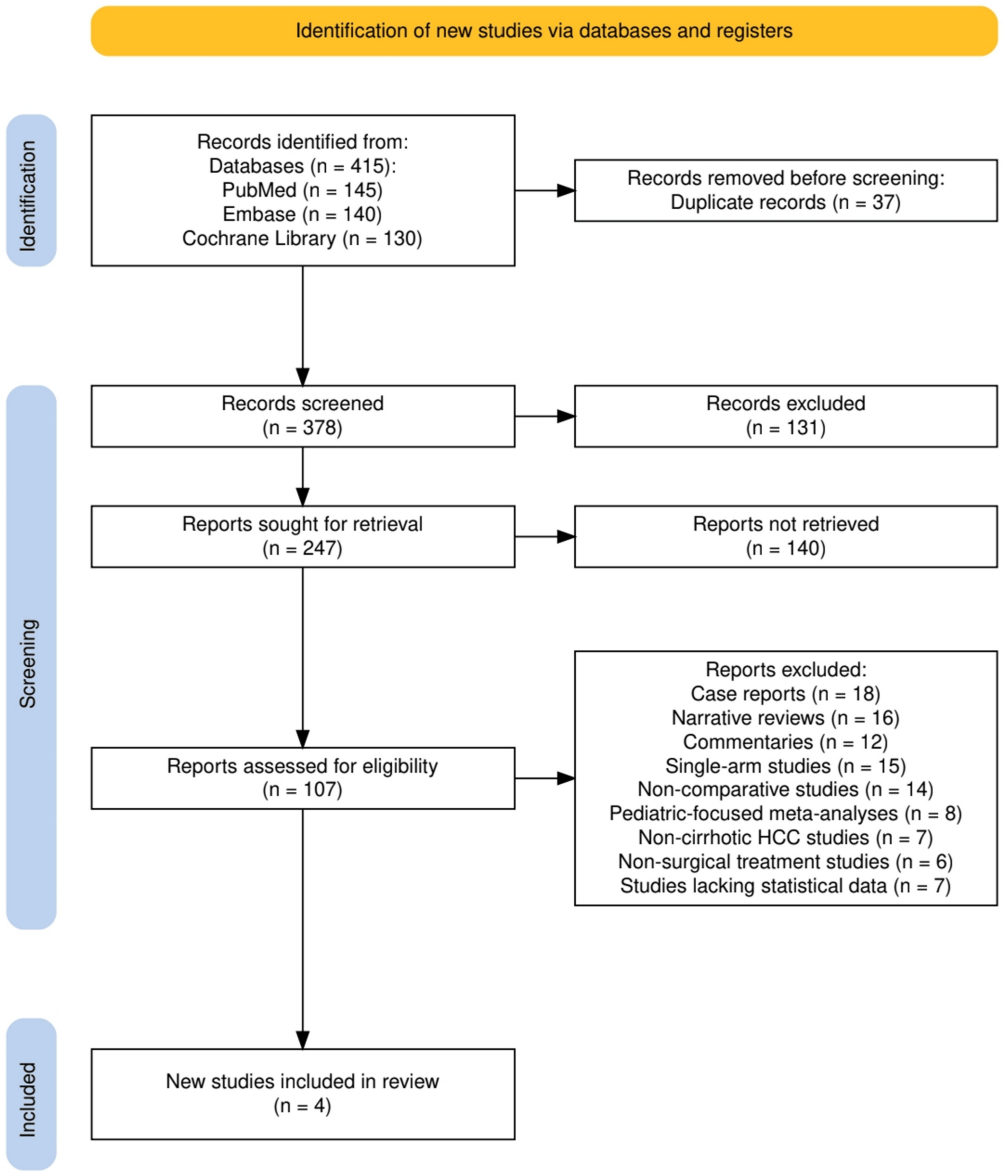
The PRISMA flowchart represents the study selection process. PRISMA: Preferred Reporting Items for Systematic Reviews and Meta-Analyses

The study selection process is illustrated in Figure 1, which presents the PRISMA flow diagram used to outline the inclusion and exclusion pathway for this systematic review. A total of 415 records were identified through database searches, including 145 from PubMed, 140 from Embase, and 130 from the Cochrane Library. After removing 37 duplicate records, 378 studies remained for title and abstract screening, of which 131 were excluded for not meeting preliminary relevance criteria. The full texts of 247 articles were sought, but 140 could not be retrieved, leaving 107 reports for full-text eligibility assessment. Of these, 103 studies were excluded based on predetermined criteria such as being case reports (n = 18), narrative reviews (n = 16), commentaries (n = 12), single-arm studies (n = 15), or lacking comparative outcomes (n = 14). Other exclusions included pediatric-only meta-analyses (n = 8), non-cirrhotic HCC studies (n = 7), non-surgical treatment studies (n = 6), and reports lacking sufficient statistical data (n = 7). Ultimately, four high-quality meta-analyses met all eligibility criteria and were included in the final review.

Study Selection Process

The study selection process is summarized in Table 1, which outlines the core characteristics of the four meta-analyses included in this systematic review. All selected studies met the predefined inclusion criteria, focusing on adult patients with HCC and underlying cirrhosis, and provided direct comparisons between LT and LR. The included studies varied in sample size, ranging from tens of thousands of patients across dozens of studies to high-level syntheses combining previous meta-analyses. Most studies focused on patients within or close to the Milan criteria, although some extended to broader HCC populations. Despite differences in methodology and follow-up duration, all studies consistently reported superior long-term OS and DFS outcomes for LT, along with lower recurrence rates compared to LR. In addition, several studies addressed key nuances such as early-stage versus advanced disease, ITT analyses, and the role of salvage transplantation, further strengthening the evidence base for this review.

**Table 1:** A summary of clinical outcomes, including survival rates, recurrence rates, and postoperative complications, for patients undergoing LT versus LR. HCC: hepatocellular carcinoma, LT: liver transplantation, LR: liver resection, OS: overall survival, DFS: disease-free survival, RFS: recurrence-free survival, HR: hazard ratio, CI: confidence interval, P: p-value, OR: odds ratio, ITT: intention-to-treat

Study (author, year)	Study design	Population (sample size)	Inclusion criteria / tumor stage	Type of cirrhosis	Intervention (LT)	Comparison (LR)	Follow-up duration	Overall survival (OS)	Disease-free survival (DFS)	Recurrence rate	Key findings / notes
Koh et al., 2022 [9]	Meta-analysis	18,421 patients (35 studies)	HCC within Milan criteria	Not specified explicitly, assumed due to Milan criteria	Liver Transplantation	Liver Resection	Varies across included studies	LT superior (HR = 1.44; 95% CI: 1.14–1.81; P<0.01)	LT superior (HR = 2.71; 95% CI: 2.23–3.28; P<0.01)	3x higher in LR than LT	Comparable OS in uninodular HCC; regional & temporal differences; enhanced surveillance improves LR outcomes
Xu et al., 2014 [10]	Meta-analysis	17 studies (sample size not stated)	Patients with HCC, tumor stage not specified	Assumed cirrhosis, not clearly stated	Liver Transplantation	Liver Resection	1, 3, and 5 years	LT had higher 3-year (OR = 1.12) and 5-year survival (OR = 1.84)	LT superior at 1 yr (OR = 1.72), 3 yrs (OR = 3.75), 5 yrs (OR = 5.64)	Lower recurrence implied in LT	LT had higher morbidity & mortality at 1 year; long-term survival & tumor-free survival favored LT
Drefs et al., 2024 [11]	Meta-analysis	19,804 patients (63 studies)	HCC patients, not restricted to Milan criteria	Not clearly specified, assumed cirrhotic background due to HCC context	Liver Transplantation	Liver Resection	5-year outcomes emphasized	LT = 64.83%, LR = 50.83% (OR = 1.79, p < 0.001)	LT = 70.20%, LR = 34.46% (OR = 5.32, p < 0.001)	Significantly lower in LT	LT outperforms LR in 5-year OS and RFS; LR outcomes improved over decades
Martinino et al., 2024 [12]	Meta-analysis of meta-analyses	10 meta-analyses (exact patient count not stated)	HCC, includes early-stage and ITT populations	Not specified directly, assumed due to HCC context	Liver Transplantation	Liver Resection	5-year outcomes analyzed	LT superior: OR = 0.79 (95% CI: 0.67–0.93); Early HCC = 0.63 (95% CI: 0.50–0.78)	LT superior: OR = 0.44 (95% CI: 0.25–0.75); Early HCC = 0.60 (95% CI: 0.39–0.92)	No difference between salvage LT and primary LT	LT shows better 5-year OS and DFS overall and in early HCC; salvage LT = primary LT

Quality Assessment

The quality assessment of the included studies, as given in Table 2, was conducted using the AMSTAR 2 tool, which evaluates the methodological rigor of systematic reviews and meta-analyses. As outlined in the quality assessment table, three of the four studies were rated as high quality with a low risk of bias, while one study was rated as moderate quality due to methodological limitations. The most recent meta-analyses demonstrated strong adherence to best practices, including protocol registration, duplicate data extraction, assessment of risk of bias, and publication bias analysis. These studies also featured comprehensive search strategies and robust statistical analyses, enhancing the reliability of their findings. One older study, while valuable, showed a moderate risk of bias due to limited reporting on protocol transparency and bias assessment methods. Overall, the included studies exhibited strong methodological consistency, contributing to the credibility and robustness of the conclusions drawn in this systematic review.

**Table 2:** A comparison of intraoperative, early postoperative, and long-term complications observed in patients undergoing LT versus LR. LT: liver transplantation, LR: liver resection

Study (author, year)	Study design	Quality assessment tool used	Key quality domains	Risk of bias	Overall quality rating	Notes
Koh et al., 2022 [9]	Meta-analysis	AMSTAR 2	Protocol registration, duplicate data extraction, risk of bias, publication bias	Low	High	Well-conducted with modern methods and large sample size; met most AMSTAR 2 criteria.
Xu et al., 2014 [10]	Meta-analysis	AMSTAR 2	Inclusion criteria, statistical heterogeneity, risk of bias, sensitivity analysis	Moderate	Moderate	Older study with limited reporting on protocol registration and bias assessment.
Drefs et al., 2024 [11]	Meta-analysis	AMSTAR 2	Time-trend analysis, comprehensive search, risk of bias, statistical robustness	Low	High	Robust long-term trends assessed; comprehensive and recent methodology.
Martinino et al., 2024 [12]	Meta-analysis of meta-analyses	AMSTAR 2 (for umbrella reviews)	Overlapping data synthesis, consistency, quality of included meta-analyses	Low	High	High-level synthesis of previous meta-analyses; consistency confirmed with clustering.

Discussion

This systematic review synthesized findings from four high-quality meta-analyses, encompassing over 55,000 patients, to compare long-term survival and recurrence outcomes between LT and LR in HCC patients with underlying cirrhosis. Across all studies, LT consistently demonstrated superior long-term OS and DFS compared to LR. For instance, Koh et al. [9] reported a significantly higher OS (HR = 1.44) and DFS (HR = 2.71) in favor of LT, with a threefold higher recurrence rate observed in the LR group. Xu et al. [10] found LT to be associated with improved three- and five-year survival outcomes, despite a higher one-year morbidity and mortality. Drefs et al. [11] highlighted LT's five-year OS and DFS superiority, with survival rates of 64.83% and 70.20%, respectively, compared to 50.83% and 34.46% for LR. Martinino et al. [12], in a meta-analysis of meta-analyses, confirmed LT's advantage even in early-stage HCC and ITT analyses, showing better outcomes than LR without significant differences between primary and salvage LT. Collectively, these findings reinforce the role of LT as the superior treatment option for long-term oncologic outcomes in appropriately selected HCC patients.

The findings of this review carry significant implications for clinical decision-making in the management of HCC with cirrhosis. The consistent advantage of LT over LR in terms of both survival and recurrence outcomes underscores the value of transplantation as the gold standard for eligible patients [13], particularly those within the Milan criteria or with early-stage disease. However, the practical application of LT is often limited by factors such as donor organ availability, waiting list dropout, and postoperative immunosuppression risks [14]. These results suggest that in settings with access to timely transplantation, LT should be prioritized, especially for patients with multifocal disease or poor liver reserve. Meanwhile, LR remains a viable initial option for patients with resectable tumors, preserved liver function, and no immediate access to transplantation, with the potential for salvage LT in the event of recurrence [15]. In addition, the enhanced outcomes observed in LR cohorts with structured surveillance protocols highlight the importance of rigorous follow-up and early detection strategies post-resection. These findings advocate for a personalized, risk-stratified approach to treatment selection, optimizing both curative intent and long-term survival in patients with HCC and cirrhosis [16].

The findings of this systematic review are largely in agreement with the existing literature and major clinical guidelines, including those from the American Association for the Study of Liver Diseases (AASLD) [17] and the European Association for the Study of the Liver (EASL) [18]. Both guidelines emphasize LT as the preferred treatment for patients with early-stage HCC within the Milan criteria and decompensated cirrhosis, citing superior survival and lower recurrence rates. The meta-analyses included in this review support these recommendations, consistently demonstrating improved long-term outcomes with LT over LR. Notably, the review by Martinino et al. [12] reinforces the notion that even salvage LT, following initial resection, may yield comparable outcomes to primary LT, aligning with the emerging literature advocating for a sequential resection-then-transplant strategy in resource-constrained environments. However, some discrepancies exist regarding the benefit of LT over LR in specific subgroups, such as patients with solitary tumors and good liver function (typically characterized by preserved hepatic synthetic function, absence of clinically significant portal hypertension, and Child-Pugh class A status), where the survival advantage of LT becomes less pronounced, as suggested in Koh et al.'s [9] analysis. These nuances underscore the importance of individualized treatment planning based on tumor burden, liver function, and transplant feasibility.

The superior long-term outcomes associated with LT over LR can be attributed to several biological and pathophysiological factors [19]. First and foremost, LT offers the dual advantage of eradicating both the tumor and the underlying cirrhotic liver, which is a fertile ground for de novo tumor formation [20]. By contrast, LR removes only the visible lesion, leaving the diseased liver parenchyma intact, thereby predisposing patients to a higher recurrence risk, particularly in the presence of microvascular invasion or satellite nodules. Moreover, cirrhosis is associated with chronic inflammation, angiogenesis, and immune dysregulation, all of which contribute to hepatocarcinogenesis and tumor progression. Transplantation effectively resets the hepatic microenvironment, potentially improving immunologic surveillance and reducing carcinogenic stimuli [21]. In addition, resection may be associated with surgical stress-induced immunosuppression, further facilitating recurrence in high-risk individuals. These mechanistic insights help explain why DFS and OS are consistently higher in transplant cohorts, reinforcing the biological rationale for favoring LT in eligible HCC patients.

Subgroup analyses within the included studies offer important insights that can refine clinical decision-making. For patients meeting the Milan criteria, typically early-stage HCC with limited tumor burden, LT consistently yields superior long-term survival and recurrence outcomes compared to resection [22]. However, in cases of solitary tumors and well-preserved liver function, such as uninodular disease, the survival difference between LT and LR narrows, as noted in Koh et al.'s meta-analysis [9]. This highlights a potential role for LR as an initial treatment in select patients, with salvage LT reserved for recurrence. Moreover, ITT analyses underscore the real-world complexities of LT, including waitlist dropout, which can affect outcomes. Geographically, differences were observed between regions; LT showed a greater survival benefit over LR in North America and Europe, whereas this disparity was less pronounced in Asian cohorts, possibly reflecting regional variations in surgical practices, selection criteria, and access to transplantation. For instance, centers in high-income countries may have better-established organ procurement systems, standardized post-transplant care, and broader access to multidisciplinary teams, while some Asian centers may emphasize advanced surgical techniques and structured resection protocols as a first-line approach. Temporally, outcomes for LR have improved significantly over the decades, narrowing the gap between the two modalities in more recent studies, as shown by Drefs et al. [11], suggesting that advancements in perioperative care and surgical techniques have elevated LR outcomes in modern practice.

Institutional factors, particularly the rigor of postoperative surveillance, appear to influence survival outcomes significantly. Koh et al. [9] observed that in cohorts receiving enhanced surveillance following LR, the overall survival difference between LR and LT diminished, indicating that meticulous follow-up and early detection of recurrence can partially offset the higher risk associated with resection. This emphasizes the importance of implementing structured surveillance protocols in centers where LR is preferred or more accessible. Additionally, disparities in donor organ availability across regions remain a major limitation to the broader application of LT. High-income countries typically maintain more robust organ procurement systems, while many low- and middle-income countries (LMICs) face challenges in donor supply, transplant infrastructure, and healthcare financing, often making LR the only feasible option [23]. Variability in institutional expertise, surgeon experience, and access to immunosuppression management further contribute to outcome differences. These real-world considerations highlight the necessity of aligning treatment strategies not only with clinical guidelines but also with local healthcare capacities and patient-specific circumstances.

This systematic review possesses several notable strengths that enhance its validity and clinical relevance. The inclusion of recent and high-quality meta-analyses, including a meta-analysis of meta-analyses, ensures a broad and up-to-date synthesis of available evidence. A comprehensive search strategy was employed, drawing from large-scale studies that collectively analyzed data from tens of thousands of patients, providing robust statistical power and generalizability. Moreover, the review highlights diverse clinical endpoints such as OS, DFS, and recurrence, offering a multidimensional assessment of treatment efficacy. Nonetheless, certain limitations must be acknowledged. The findings rely exclusively on secondary data, which may be subject to publication bias, inconsistencies in reporting, and methodological heterogeneity across the included studies. The presence of overlapping patient data in multiple meta-analyses could influence pooled effect estimates, while variations in follow-up duration, surgical expertise, and institutional protocols introduce further complexity. In addition, key patient-level variables, such as liver function, AFP levels, and comorbidities, were inconsistently reported or omitted entirely, limiting the ability to perform subgroup stratification and personalized analysis. These constraints should be considered when interpreting the conclusions of this review.

The findings of this review offer clear clinical guidance by reinforcing LR as the preferred treatment modality for eligible patients with hepatocellular carcinoma, particularly those within the Milan criteria or with underlying decompensated cirrhosis. Clinicians should consider patient-specific factors such as liver function, tumor characteristics, and access to transplant facilities when formulating individualized treatment plans. In patients with preserved hepatic reserve and solitary tumors, LR may serve as a suitable initial intervention, with salvage transplantation reserved for recurrence, especially in settings where timely organ allocation is uncertain [24]. From a policy and resource perspective, the broader implementation of LT is hindered by organ scarcity, limited surgical capacity, and economic constraints, particularly in LMICs. These disparities highlight the need for equitable organ allocation systems, investment in transplant infrastructure, and region-specific guidelines that account for healthcare limitations. Until these systemic barriers are addressed, LR will continue to play a critical role in HCC management, especially in resource-constrained environments.

Despite the robust evidence supporting the superiority of LT over LR in selected HCC patients, several important research gaps remain. Future studies should focus on well-designed prospective trials directly comparing LR and LT in specific subgroups, such as patients with borderline liver function or intermediate-stage tumors. The role of molecular and genetic biomarkers in predicting recurrence and treatment response also warrants exploration, as personalized medicine continues to evolve. In addition, more data are needed on patient-reported outcomes, quality of life, and functional recovery following each procedure, as these factors are critical to comprehensive decision-making but remain underreported in the current literature. Research evaluating the cost-effectiveness of salvage LT strategies and long-term surveillance protocols could further optimize treatment pathways, particularly in settings with limited resources or long transplant waitlists.

## Conclusions

This systematic review demonstrates that LT offers superior long-term survival and significantly lower recurrence rates compared to LR in patients with HCC and underlying cirrhosis, particularly those within the Milan criteria. While resection remains a valuable option in select cases, especially where transplantation is not readily accessible, the evidence overwhelmingly supports LT as the gold standard in eligible patients. These findings underscore the importance of individualized treatment planning and highlight the need for health systems to improve access to transplantation, optimize surveillance strategies, and invest in research that advances precision in liver cancer care.
